# Malignant Gliomas as Second Neoplasms in Pediatric Cancer Survivors: Neuropathological Study

**DOI:** 10.1155/2018/4596812

**Published:** 2018-04-01

**Authors:** Ewa Izycka-Swieszewska, Ewa Bien, Joanna Stefanowicz, Edyta Szurowska, Ewa Szutowicz-Zielinska, Magdalena Koczkowska, Dawid Sigorski, Wojciech Kloc, Wojciech Rogowski, Elzbieta Adamkiewicz-Drozynska

**Affiliations:** ^1^Department of Pathology and Neuropathology, Medical University of Gdańsk and Department of Pathomorphology, Copernicus PL Hospitals, Gdańsk, Poland; ^2^Department of Pediatrics, Hematology and Oncology, Medical University of Gdańsk, Gdańsk, Poland; ^3^Second Department of Radiology, Medical University of Gdańsk, Gdańsk, Poland; ^4^Department of Oncology and Radiotherapy, Medical University of Gdańsk, Gdańsk, Poland; ^5^Department of Biology and Genetics, Medical University of Gdańsk, Gdańsk, Poland; ^6^Clinical Department of Oncology & Immunooncology, Hospital with Oncology Center MSW, Olsztyn, Poland; ^7^Department of Neurosurgery, Copernicus Hospital, Gdańsk, Poland; ^8^Warmia-Mazury University, Olsztyn, Poland; ^9^Department of Clinical Oncology, Magodent, Warsaw, Poland

## Abstract

This study presents a unique series of malignant supratentorial gliomas in children previously cured from non-CNS primary cancer. On neuroimaging these tumors were not specific, so the patients were suspected of cerebral recurrence of their primary neoplasm: leukemia in four children and sarcoma in one child. Histologically, the group contained four glioblastomas and one anaplastic astrocytoma. Three patients underwent neurosurgical resection, while the other two underwent stereotactic diagnostic biopsy only. Despite combined oncological treatment, four children died during 20 months, and only one glioblastoma patient continued to live for another twelve years. Microscopically, the neoplasms consisted of small cells with some morphologic features of astrocytic lineage, having scanty or prominent processes. Microvascular proliferation and focal or diffuse necrosis were encountered in four cases. The GFAP reactivity in neoplastic cells was low or nil, together with the expression of Olig2, vimentin, and nestin. In two cases a subpopulation of synaptophysin-positive cells was present. Molecular immunohistochemical profiling revealed the expression of phosphorylated forms of PI3Kp110 and AKT, in parallel to a strong PTEN and p53 positivity. The tumors were of IDH1R132H-wild type and immunoreactive for ATRX, HER3, and EGFR. Secondary malignant gliomas in pediatric cancer survivors pose a diagnostic challenge. The present study shows that these tumors are of IDH wild type, PI3K/AKT-activated, having no PTEN and EGFR mutations. Therefore, the biopsy of brain tumors in such patients is crucial both for accurate diagnosis and material preservation for molecular typing.

## 1. Introduction

The survival rate of the childhood cancer patients has significantly improved because of the progress in treatment methods. However, the intensification of therapy at young age causes many long-term complications. One of the serious problems in pediatric oncology is a several-fold increase of secondary cancer risk, in comparison to the general population [1–3]. The most common childhood malignancy is acute lymphoblastic leukemia (ALL), followed by solid tumors such as brain neoplasms, neuroblastoma, nephroblastoma, and rhabdomyosarcoma. Conversely, the secondary malignancies in children are represented mainly by acute nonlymphoblastic leukemias, brain tumors, and thyroid cancer. Multiple factors may increase the risk of developing these secondary malignancies in childhood cancer survivors with both intrinsic and environmental aspects [2–4]. They are probably caused by the fact that ionizing radiation, multimodal chemotherapy, and immunosuppression interfere with the ongoing developmental and maturation processes. Some children can develop metachronic neoplasms either due to genetic syndromes such as neurofibromatosis, Li-Fraumeni, and Beckwith-Wiedemann syndromes or through unsettled, most probably genetic, susceptibility [1–3, 5].

The relation between the child's age, tumor histology, and the latent period for the secondary neoplasm development has been confirmed [1, 5]. Radiation therapy of the CNS region is associated with an increased risk of any subsequent brain tumor, especially gliomas (secondary malignant glioma, SMG) [4, 6, 7]. Primary pediatric brain tumors show age and site dependency, arising from topographically discrete progenitor cells. High grade pediatric gliomas are rather infrequent in general population; they comprise histological categories such as glioblastoma, anaplastic astrocytomas, anaplastic oligodendrogliomas, and diffuse intrinsic pontine glioma. Their gene expression and DNA methylation signatures are distinctive, often reflecting a developmental context [8–10]. SMGs arise as a late side effect of ALL or primary brain tumors treatment, usually developing within the irradiation field in subsequent 10 years. ALL patients typically receive chemotherapy and craniospinal radiotherapy and achieve up to 90% cure rate, but with a significant risk of a secondary cancer [11, 12]. The clinical presentation of SMG is not specific, and also radiologically it can be suggestive of a primary cancer recurrence, metastasis, inflammatory lesion, or radionecrosis. Despite significant advances in neuroimaging, a precise differential diagnosis of these lesions requires histopathological evaluation [10, 13, 14]. Secondary high grade gliomas, as an unusual subgroup of pediatric brain tumors, have been seldom elaborated neuropathologically. The molecular data on these neoplasms is scanty. They are not well characterized due to their low incidence and poor availability of tissue material for the analyses.

## 2. Material and Methods

The study was performed on archival tissue material obtained from the patients treated in the Department of Pediatrics, Hematology & Oncology, Medical University of Gdańsk between 1994 and 2010. Within this period, five patients with brain tumors as the secondary primary malignancy were diagnosed. The written permission for medical research was obtained from their parents at the moment of treatment initiation. Archival, routinely processed (formalin-fixed, paraffin-embedded) tumor tissue samples were used in the study. Neuropathological reexamination of all available tumor sections was also performed. The analyzed tissue material came from four stereotactic biopsies and four tumor resections and was mostly scant and in one case eligible for only a few immunostains.

The selected tumor sections were immunophenotyped with standard procedures according to the manufacturer's recommendations. Phosphospecific antibodies protocols were carried out specifically with an overnight incubation at 4°C. Dako EnVision Systems served for visualization, with diaminobenzidine used as the chromogen. Appropriate positive and negative controls were carried out in parallel. The following detecting antibodies were used: alpha smooth muscle actin (1A4), CD34 (QBEnd 10), CD56 (123C3), CD99 (12E7), chromogranin (pab), cytokeratin (AE1∖AE3), desmin (D33), MyoD1, EMA (E29), GFAP (pab), vimentin (V9), LCA (M0701), Ki67 (MIB1), synaptophysin (S38), p53 protein (clone 318-6-11), TdT, OCT3/4, and bcl2 (all by DAKO, Denmark, ready-to-use). Other antibodies were also employed, with their specific protocols, against nestin (10c2: sc-23927) and Olig2 (C-17) (both by Santa Cruz Biotechnology, USA); EGFR (clone EGFR.113), HER3, and PTEN (NCL-PTEN) (all three by Novocastra, GB); IDH1 (R132H) by Dianova; ATRX (0537) and MGMT (MT3.1) by Abcam. Moreover, phosphospecific antibodies against p-PI3Kp110 (pab, 1 : 100 Santa Cruz Biotechnology CA, USA), pAKT (Ser473, 1 : 25, 736E11), p-mTOR (Ser 2448, 1 : 50 clone 49F9), and p-70S6K (Thr 389, mAb, 1 : 100) by Cell Signaling Technology, MA, USA, were also applied. The pattern of protein expression (membranous, cytoplasmic, nuclear) and its intensity (negative, low, strong) within the neoplastic cells were microscopically assessed individually for every antibody (microscope Olympus BX53). Assessment of PI3K/AKT/mTOR pathway, as well as EGFR and HER3, was carried out with our own scale [15]. The Ki67 index was counted in 500 neoplastic cells in “hot spots.” MGMT expression was scored as low or high, based on the percentage of positive cells (30% as a cut-off point) [16]. P53 was interpreted as positive when more than 20% cells showed strong nuclear staining.

For DNA extraction, 5-*μ*m sections were cut from paraffin blocks of three available tumor samples, and genomic DNA was isolated using the standard protocol with proteinase K digestion, phenol-chloroform extraction, and ethanol precipitation. The somatic status of* EGFR* (exons 2–7 and 18–21) was analyzed using a polymerase chain reaction (PCR) and direct sequencing. Specific primers were designed using the Primer3 web tool (http://frodo.wi.mit.edu/primer3), and their sequences and PCR cycling parameters are available on request. The obtained sequences were compared with reference transcript sequences available online (http://www.ensembl.org,* EGFR*: ENST00000275493), using Sequencer v.4.7 (Gene Codes Corporation, USA).

## 3. Results

**Figure 1:**
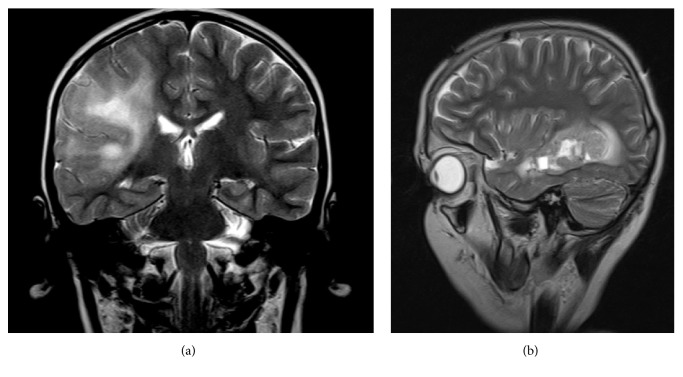
Magnetic resonance imaging of chosen gliomas. (a) Contrast-enhancing heterogenous diffuse infiltrating tumor involving two lobes with diffuse edema (P4, coronary, postcontrast T2), (b) recurrent well-demarcated glioblastoma surrounded with irregular edema (P5, sagittal, postcontrast T2).

The examined group consisted of five patients, whose clinical characteristics are summarized in Table 1. Four children at the age of 2.5–9 years (three boys and one girl, P1–P4) were primarily diagnosed with ALL, two low-risk and two high-risk cases. The children underwent a full three-year-long hematologic treatment including chemotherapy with BFM-90 regimen. The applied CNS irradiation dose was prophylactic in three children and therapeutic in one, achieving complete remission of the disease. In the ALL children, the secondary brain tumor developed 3–6.5 years after the conclusion of leukemia therapy. They showed acute neurologic symptoms: seizures and/or rapidly progressing paresis. A detailed clinical description of the three patients mentioned above has been previously published [11]. Radiologically, all of these brain tumors were supratentorial and contrast-enhancing. Three were diffusely infiltrating with the involvement of at least two cerebral lobes, and only one was well demarcated (Figures 1(a) and 1(b)). One tumor contained prominent hemorrhagic fraction. In three children diagnostic stereotactic biopsy was performed first, followed by a subtotal surgical resection in one child and total in another one. One patient underwent a partial tumor resection without a prior biopsy. Histologically, three glioblastomas and one anaplastic astrocytoma were diagnosed. The differential diagnosis in all cases included a cerebral relapse of primary cancer, malignant gliomas, and other pediatric tumors. At the intraoperative examination from clotty material, case P1 was erroneously recognized as leukemic infiltration. After the final histopathological diagnosis the child was reoperated on. Despite the introduced complex neurooncological treatment for high grade gliomas, all post-ALL children did not achieve remission and succumbed to the tumor progression within 5 to 19 months.

The fifth patient (case P5) was a boy diagnosed with a right maxillary rhabdomyosarcoma (RMS) at the age of 11. He completed treatment with the CWS-96 protocol of chemotherapy with local maxillary region irradiation (48 Gy) and remained in complete remission for two years. A control brain CT in this asymptomatic boy detected a well-demarcated contrast-enhancing 2 cm tumor in the left temporal lobe. This case was initially diagnosed as an RMS metastasis, based on scanty biopsy material (phenotyping was infeasible). That discrepancy caused a two-month delay in proper treatment and led to a repeated biopsy before the resection of glioblastoma. The gross total excision was followed by a local radiotherapy and temozolomide regimen. The patient experienced a complete remission lasting 6.5 years till the local glioma recurrence, consequently completely excised. After a consecutive second line chemotherapy, a complete remission lasting 3.5 years was achieved. However, the next local chemoresistant progression caused the patient's death in the twelfth year of glioblastoma course.

**Figure 2:**
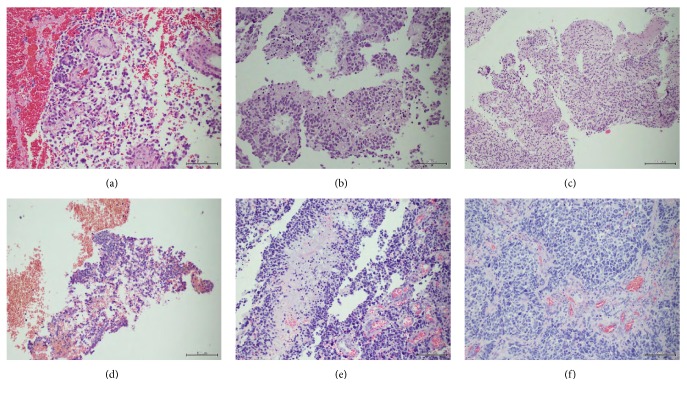
Histopathological pictures of examined tumors, showing malignant small cell glial neoplasms with focal necrosis and microvascular proliferation. (a) Case P1, tumor resection (HE, 200x), (b) case P2, biopsy (HE, 200x), (c) case P3, partial tumor resection (HE, 100x), (d) case P4, biopsy (HE, 100x), (e) case P5, repeated biopsy (HE, 200x), (f) case P5, tumor resection at recurrence (HE, 200x).

Histologically, four tumors (P1, 2, 4, 5) were cellular rich, composed of small cells with short processes and round blue cells. The neoplastic cells focally formed perivascular pseudorosettes. P3 tumor had a loose structure with stellate cells. Recurrent P5 glioblastoma showed a prominent increase of cellular anaplasia and proliferation. It had a lobular arrangement of neoplastic tissue underlined with pathological vessels. Mitotic and apoptotic activity were visible in all gliomas with a necrosis including a pseudopalisading type in four of them. Microvascular proliferation with vascular wall thickening and multiluminal structure formation characterized three cases (Figures 2(a)–2(f)). Thrombosis and hemorrhages were also present in two tumors.

**Figure 3:**
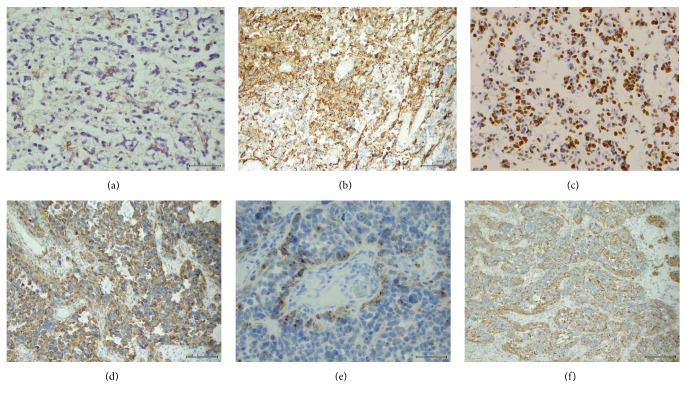
Details of neoplastic immunophenotype: (a) GFAP-negative tumor cells and immunopositive reactive astrocytes (P3, GFAP, 400x), (b) moderate GFAP staining in neoplastic cells (P1, GFAP, 200x), (c) high proliferative index in glioblastoma (P3, Ki-67, 200x), (d) strong nestin expression in recurrent tumor (P5r, nestin, 200x), (e) perivascular subpopulation of synaptophysin-reactive cells (P2, synaptophysin, 400x), (f) intense vimentin staining in recurrent GFAP-negative glioblastoma (P5r, vimentin, 200x).

Immunohistochemistry (Figures 3(a)–3(f)) disclosed positivity of neoplastic cells for GFAP in four gliomas. However, reactivity in three tumors was low and unevenly distributed. The neoplastic cells showed strong vimentin, moderate nestin, and low Olig2 reactivity. Proliferative index (Ki-67) ranged from 15 to 50% in the analyzed hot spots. In addition, a local expression of synaptophysin was observed in perivascular cells in case P2 and in recurrent glioblastoma P5. P5 regrowing tumor revealed almost complete loss of GFAP, diffuse nestin staining, and de novo focal EMA reactivity in a dot-like pattern, compared to the primary one. In all of the examined tumors, other neural, myogenic, epithelial, and hemato-lymphocytic markers were negative.

**Figure 4:**
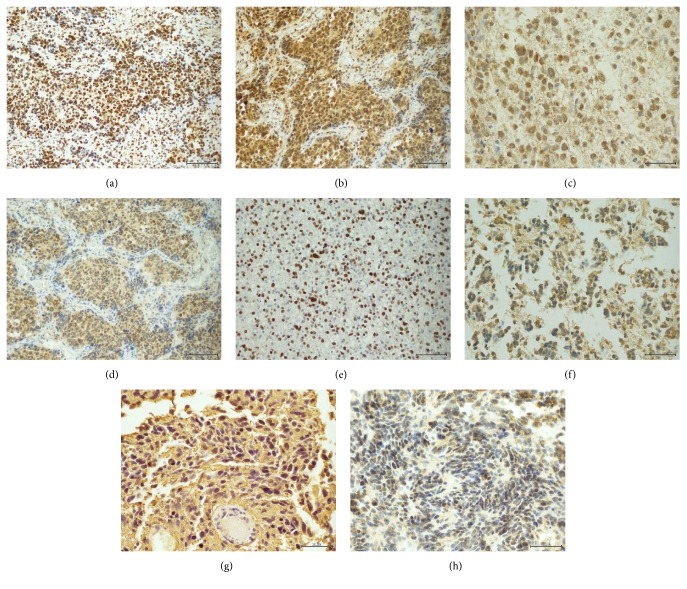
Molecular findings assessed immunohistochemically: (a) strong nuclear PTEN reactivity (P1, PTEN, 200x), (b) strong PI3K expression (P5rec, PI3K, 200x), (c) cytoplasmic/nuclear pAKT immunoreactivity (P1, pAKT, 400x) (d) p-mTOR cytoplasmic/nuclear expression within the neoplastic cells (P5rec, p-mTOR, 200x), (e) nuclear p53 staining within 60% of glioma cells (P2, p53, 200x), (f) moderate cytoplasmic/membranous EGFR expression (P3, EGFR, 400x), (g) strong cytoplasmic HER3 reactivity (P1, HER3, 400x), (h) high level MGMT staining (P3, MGMT, 200x).

Molecular pathology profiling revealed PI3K/AKT/mTOR signaling pathway molecules expression in four cases suitable for examination. Strong nuclear PTEN characterized all of the analyzed gliomas. Strong cytoplasmic expression of a phosphorylated form of PI3Kp110 and cytoplasmic and nuclear phospho-AKT were also detected. Moderate cytoplasmic phospho-mTOR and pS70K staining occurred only in primary and recurrent glioblastoma P5. A cytoplasmic/membranous moderate to strong immunoreactivity for EGFR and HER3 was also found in neoplastic cells of all four tumors. Mutational analysis did not reveal any EGFR mutations in the three available cases. Furthermore, the examined cases revealed p53 positivity (from 30 to 80% of cells) and retained nuclear ATRX staining. IDH-1 R132H negativity characterized all four analyzed cases. High MGMT staining intensity was observed in three tumors and low intensity in glioblastoma with a prolonged course (Table 2, Figures 4(a)–4(h)).

## 4. Discussion

The development of secondary neoplasms creates a serious adverse event in children cured of cancer. The clinical and epidemiological analysis of 642 secondary cancers in the patients previously treated for ALL showed that the most common are hematologic malignancies (about 54%), brain tumors (21%), and thyroid cancers [3, 5, 17]. Secondary malignant gliomas, usually of glioblastoma type, occur in about 2% of different cancer patients 10–15 years after radiotherapy. The second most frequent are meningiomas associated with low radiation doses and long latency, up to 20 years; secondary low-grade gliomas, embryonal tumors, and sarcomas [4–7] are rarely seen. The main postulated cause of SMG is the irradiation influence on the viability and function of neural stem cells [7, 18]. Irradiation induces DNA damage, genetic alterations, and karyotypic instability and affects cell signaling and gene expression [5]. In recent years CNS radiotherapy has been modified in order to minimize its cancerogenic effect and delayed cognitive deficits in young patients [1]. It is worth mentioning that in the most extensive review summarizing clinical aspects of 176 cases published in the years 1960–2013 the relation between irradiation and brain tumors as secondary malignancy was not unequivocally confirmed [13]. It seems that chemotherapy with alkylating agents such as nitrosourea, temozolomide, and methotrexate acts as a cocancerogenic factor [3, 12].

Pediatric SMGs are clinically quite well characterized, while neuropathological data on them is sparse as only few tissue and molecular studies have been carried out [17, 19–21]. It happens that SMGs are diagnosed solely radiologically, without tumor sampling, or undergo only a small stereotactic biopsy. The most extensive study by Romeike et al. analyzed nine pediatric SMG cases, Brat et al. [17] examined four pediatric and five adult secondary gliomas. Donson et al. [20] with his 5 cases supported the previous reports, and furthermore there are few single case reports available. Our study was carried out on five pediatric hemispheric SMGs, including four cases which concurred with the definition of radiation-induced gliomas [11, 13]. They arose after a latent period after CNS radiation during leukemia treatment, with the average interval of 8-9 years, similarly to the previous observations [5, 7, 13]. Radiologically, three tumors of our series showed diffuse infiltration and occupied more than two cerebral lobes. Only an initially asymptomatic glioblastoma in an RMS survivor developed outside the irradiation field. Very poor prognosis in SMGs is caused by the difficulty of an adequate and sufficient resection and a reduced reuse of radiotherapy. Only single reports of durable survival of SMG patients were published [5, 22, 23]. One of our patients experienced a uniquely long follow-up, being totally resected twice, and treated with temozolomide. In this case we should also consider a possibility that this late local recurrence (after 6.5 years) was a radiotherapy-induced secondary glioblastoma after the primary glioma therapy. The rest of the children with incomplete resection or biopsy only died due to a glioma progression, up to 20 months since the diagnosis, despite multimodal therapy. Accordingly, in children, gross and radiological total tumorectomy constitutes the strongest predictor of survival [14, 23].

Histologically, the presented tumors were composed of small undifferentiated, fusiform, or stellate cells and contained focal necroses and microvascular proliferation. SMGs described in the literature confirm the features described above, reminiscent of small cell glioblastoma with high proliferation index, ranging up to 67% [19]. In one glioma with recurrences, progress of cellular atypia occurred in its regrowth. Immunophenotype of our series, except for one case, showed low positivity for GFAP, evident nestin expression, and focal synaptophysin staining in two tumors. Descriptions of dedifferentiated SMGs, even GFAP-negative or gliomas with neural phenotype, are noted in the literature [19]. These findings correspond to glioblastoma heterogeneity proven with cDNA and gene expression studies, dividing them into subgroups (neural, proneural, mesenchymal, and classic) differing also at the level of protein expression.

Glioblastoma, typically affecting older adults, can develop de novo, and less frequently (up to 10%) as a secondary tumor progressing from lower grade astrocytomas. The latest WHO classification recognizes two basic types of glioblastomas: IDH wild type (primary, 90% of cases) and IDH-mutant (secondary) type [24, 25]. In primary glioblastoma, the most common genetic disorder is the loss of heterozygosity on chromosome 10q (70%), amplification of the* EGFR* (36%),* PTEN* mutations, and deletion of p16. In secondary glioblastomas with* IDH1* mutations,* TP53* mutation is a dominant (65%) feature, accompanied by 19q loss [24, 26–28]. In general,* EGFR* alterations occur in 50% of adult tumors comprising gene amplification, mutation, overexpression, and intragenic deletion resulting in EGFR vIII [24, 25, 29]. However, pediatric high grade gliomas constitute a heterogenous group of tumors with distinctive gene expression and DNA methylation signatures different from those seen in adults [8–10]. Recently, integrated molecular analysis revealed biological subsets of pediatric malignant gliomas with associated prognostic markers. In general, they can be divided into midline and hemispheric neoplasms, but other subclasses are also discernible [24, 30, 31]. Diffuse midline gliomas show histone mutations* H3F3A* K27, since hemispheric-G34 alterations are accompanied by* ATRX* mutations with loss of its tissue expression [21, 27, 30, 31].* IDH1* and* IDH2* mutations are very rare in pediatric astrocytomas. In children, H3/IDH wild type seems to be quite frequent (with emerging molecular subtypes), as well as a group with either low-grade or PXA-like molecular features [10, 32, 33]. Molecular genetic profiling of pediatric high grade gliomas shows frequent alteration as a gain of chromosome 1q (30%) and lower frequency of a gain of chromosome 7 and 10q loss [21, 34]. Alterations in* PI3K*,* EGFR*, and* PTEN* are less frequent in pediatric than in adult gliomas. However, overexpression of AKT is observed together with more often altered* PDGFRA *and p53 pathways. Epigenetically, almost half of pediatric HGGs show promoter methylation of* MGMT* gene, and in these cases, with the absence of other mutations, a 5-year survival reaches even 42% [9, 14]. Available data demonstrate that secondary, radiation-induced tumors seem to be more homogenous genetically than spontaneous pediatric glioblastomas [7, 20, 34].

The assessment of several molecular changes, status of methylation, or signaling pathways, as well as prognostic and predictive biomarkers, may be tested successfully with specific antibodies [25, 27–29]. In our SMG series, all tumors revealed IDH-1 R132H immunonegativity pointing to a lack of corresponding gene point mutation. All of the SMGs disclosed p53 positivity with strong nuclear staining within 30–80% of the cells, suggesting a* TP53* mutation. In addition, ATRX nuclear staining excluded corresponding gene mutation and most probably H3 mutations, since* ATRX* mutations tightly accompany H3 alterations [10, 30, 31]. The tumors showed moderate to strong expression of EGFR and HER3, but no* EGFR* mutations in genetic analysis. Moreover, they revealed immunoreactivity for PI3K/AKT signaling pathway proteins, suggesting its activation in the face of strong PTEN staining. PI3K and phospho-AKT expression characterized all tumors, while p-mTOR and pS70K staining occurred only in one glioblastoma with prolonged survival. Three of our post-ALL cases revealed high MGMT; however, its estimation standardization is limited [16]. In the previous studies on SMGs, Donson et al. [20] examined their series with FISH for* EGFR* and* PTEN* and performed immunohistochemistry and gene expression microarrays. They found no* EGFR* amplification, but* PTEN* loss, as well as elevated expression in PDGFRA and SOX10. They observed, just like in our study, widespread HER3 reactivity.* PDGFRA* amplification and 1q loss at a significantly higher frequency are also described [7, 20, 21]. In the cases published by Brat et al. [17] there was neither* EGFR* amplification nor overexpression. Moreover, they detected diffuse p53 staining, with* TP53* mutation in one, and no p16 and* PTEN* changes. The series described by Romeike et al. showed lack of EGFR expression and variable p53 reactivity in glioma cells [19]. Our analysis supports new data on molecular SMG features, complementary to the previous studies.

## 5. Conclusions

Secondary high grade gliomas in pediatric cancer survivors pose a great challenge both for the clinicians and pathologists. Biopsy of these rare tumors is required for accurate histopathological diagnoses and molecular profiling. Molecular and immunohistochemical signatures classified our series as IDH1-wild type glioblastomas. They showed altered p53 and AKT activation, but neither* EGFR* mutations nor* ATRX* and* PTEN* inactivation, so their exact subtyping is not clear. The proper characterization of SMGs is essential for identification of new effective therapeutic strategies for patients.

## Figures and Tables

**Table 1:** Clinical details of the patients.

	P1	P2	P3	P4	P5
Primary cancer	ALL low risk	ALL low risk	ALL high risk	ALL high risk	RMS right maxillary sinus

Age at primary cancer diagnosis	9,5 years	6,8 years	2,8 years	4 years	11 years

CNS infiltration at primary tumor	No	No	Yes/recurrence	Yes/at diagnosis	No

Brain RTX (total dose)	Prophylactic (12 Gy)	Prophylactic (12 Gy)	Therapeutic (24 Gy)	Therapeutic (24 Gy)	No CNS radiotherapy

Response to therapy of primary tumor	CR	CR	CR	CR	CR

Interval^*∗*^	3 years	3,5 years	6,5 years	5 years	2 years

Age at brain tumor diagnosis	15,5 years	12,3 years	12 years	12 years	15 years

Brain tumor	Glioblastoma	Glioblastoma	Glioblastoma	Anaplastic astrocytoma	Glioblastoma

Localization	Right frontoparietal	Left frontoparietal	Left temporoparietooccipital	Right frontoparietotemporal	Left temporal

SMG therapy	Biopsy, tumor resection, CHT, RTX	Tumor biopsy, CHT	Partial tumor resection, CHT	Tumor biopsy, CHT	Biopsy, repeated biopsy, tumor resection, CHT, RTX after 6 yr 2nd tumorectomy, CHT

Outcome	Death of PD 19 mth	Death of PD 5 mth	Death of PD 10 mth	Death of PD 9 mth	Death of PD 12 years

*Abbreviations*. ALL: acute lymphoblastic leukemia, RMS: rhabdomyosarcoma, CNS: central nervous system, RTX: radiotherapy, CR: complete remission, Interval^*∗*^: period between the end of the first malignancy treatment and brain tumor diagnosis, SMG: secondary malignant glioma, CHT: chemotherapy, PD: progression of disease.

**Table 2:** Immunophenotype and molecular profiling of analyzed tumors.

	P1	P2	P3	P4	P5	P5 rec
GFAP	++	+	Focal +	Focal +	Focal +	Focal +
Vimentin	+++	+++	++	++	++	+++
Synaptophysin	-	Focal +	-	-	-	Focal +
EMA	-	NA	-	NA	-	Focal +
Nestin	+	-	+	Focal +	Focal +	+++
Olig2	+	+	+	+	-	+
P53	+	+	+	NA	+	+
EGFR	++	+	++	NA	++	++
HER3	++	++	++	NA	NA	++
PTEN	+++	+++	+++	NA	+++	+++
PI3Kp110	++	++	++	NA	++	+++
pAKT	++	-	++	NA	+	+++
p-mTOR	-	-	-	NA	+	++
P70S6K	-	-	-	NA	+	++
MGMT	High	High	High	NA	Low	Low
IDH1	-	-	-	NA	-	-
ATRX	+	+	+	NA	+	+
Ki67	25%	50%	15%	NA	20%	40%

*Abbreviations*. GFAP: glial fibrillary acidic protein, EMA: epithelial membrane protein, EGFR: epidermal growth factor, rec: recurrent, NA: not available.
